# Clinical context enhances interpretation of extracorporeal treatment criteria in lithium poisoning

**DOI:** 10.1093/ckj/sfag328

**Published:** 2026-09-24

**Authors:** Álvaro Pineda-Torcuato, Antonio F Caballero-Bermejo, August Supervia, Emilio Salgado, Ruadhan O’Laoi, Carolina Sánchez, Francisca Córdoba, Neus Rodríguez Farré, Natalia Sanz-López, Héctor Hernández Ontiveros, Blanca Andrea Gallardo Sánchez, Paula Romero Gallardo, Victoria Lobo-Antuña, Benjamín Climent, Sandra Mora Azabal, Alberto Cózar-Llistó, Marina Vergara Ortiz, Mikel Urroz-Elizalde, Valle Molina Samper, Pablo Navarro Román, Xavier Canseco Suárez, Francisco J Callado Moro, Cormack Kennedy, María del Pilar Gómez Jiménez, M Àngels Gispert-Ametller, Macarena Lucila Míguez del Águila, Isabel Llopis Sanmillán, Luca Cioccari, Beatriz Martín Pérez, Ana Ramos Rodríguez, Susanna Vert García, Andrea Terron Sánchez, Jaime Laureiro Gonzalo, Guillermo Burillo-Putze, Edith Gutiérrez, Gregorio Rodríguez-Boto, Santiago Nogué-Xarau, Jordi Puiguriguer-Ferrando, Belén Ruiz-Antorán

**Affiliations:** Clinical Toxicology Unit, Emergency Department and Clinical Pharmacology, Hospital Universitario Puerta de Hierro Majadahonda, Madrid, Spain; Escuela de Doctorado, Universidad Autónoma de Madrid, Madrid, Spain; Clinical Toxicology Unit, Emergency Department and Clinical Pharmacology, Hospital Universitario Puerta de Hierro Majadahonda, Madrid, Spain; Faculty of Medicine and Health Sciences, Universidad Nebrija, Madrid, Spain; Emergency Department, Hospital del Mar, Barcelona, Spain; Functional Unit of Toxicology of the Parc de Salut del Mar, Barcelona, Spain; Toxicology Working Group of the SoCMUE (SoCMUETOx), Universitat Pompeu Fabra, Barcelona, Spain; Toxicology Working Group of the SoCMUE (SoCMUETOx), Universitat Pompeu Fabra, Barcelona, Spain; Clinical Toxicology Unit, Emergency Department, Hospital Cliníc, Barcelona, Spain; Pharmacology & Therapeutics, School of Medicine, Trinity College Dublin, Dublin, Ireland; Clinical Research Facility, St James’s Hospital, Dublin, Ireland; Clinical Toxicology Unit, Emergency Department, Hospital Cliníc, Barcelona, Spain; Toxicology Working Group of the SoCMUE (SoCMUETOx), Universitat Pompeu Fabra, Barcelona, Spain; Emergency Department, Hospital Universitario Moises Broggi Sant Joan Despí, Barcelona, Spain; Nephrology Department, Hospital Universitario Moises Broggi Sant Joan Despí, Barcelona, Spain; Emergency Department, Hospital de la Santa Creu i Sant Pau, Barcelona, Spain; Toxicology Working Group of the SoCMUE (SoCMUETOx), Universitat Pompeu Fabra, Barcelona, Spain; Emergency Department, Hospital de la Santa Creu i Sant Pau, Barcelona, Spain; Emergency Department, Hospital Universitario Severo Ochoa, Madrid, Spain; Emergency Department, Hospital Universitario Severo Ochoa, Madrid, Spain; Clinical Toxicology Unit, Internal Medicine Department, Consorcio Hospital General Universitario de Valencia, Valencia, Spain; Clinical Toxicology Unit, Internal Medicine Department, Consorcio Hospital General Universitario de Valencia, Valencia, Spain; Emergency Department, Hospital Universitario de Getafe, Getafe, Madrid, Spain; Internal Medicine Department, Hospital Universitario Ramón y Cajal, Madrid, Spain; Internal Medicine Department, Hospital Universitario Ramón y Cajal, Madrid, Spain; Clinical Pharmacology Department, Hospital Universitario La Paz, Madrid, Spain; Emergency Department, Hospital Universitario de Navarra, Pamplona, Navarra, Spain; Emergency Department, Hospital del Mar, Barcelona, Spain; Functional Unit of Toxicology of the Parc de Salut del Mar, Barcelona, Spain; Emergency Department, Hospital Uniiversitario Álvaro Cunqueiro, Vigo, Spain; Emergency Department, Hospital Universitario de Burgos, Burgos, Spain; Pharmacology & Therapeutics, School of Medicine, Trinity College Dublin, Dublin, Ireland; Clinical Research Facility, St James’s Hospital, Dublin, Ireland; Emergency Department, Hospital Universitario Juan Ramón Jiménez, Huelva, Spain; Toxicology Working Group of the SoCMUE (SoCMUETOx), Universitat Pompeu Fabra, Barcelona, Spain; Emergency Department, Hospital Universitario Doctor Josep Trueta, Girona, Spain; Emergency Department, Hospital Universitario Doctor Josep Trueta, Girona, Spain; Department of Intensive Care Medicine, Kantonsspital Aarau, Aarau, Switzerland; Department of Intensive Care Medicine, Kantonsspital Aarau, Aarau, Switzerland; Department of Intensive Care Medicine, University of Bern, Bern University Hospital, Bern, Switzerland; Emergency Department, Hospital Universitario Río Hortega, Valladolid, Spain; Emergency Department, Hospital Universitario Río Hortega, Valladolid, Spain; Toxicology Working Group of the SoCMUE (SoCMUETOx), Universitat Pompeu Fabra, Barcelona, Spain; Emergency Department, Hospital de Viladecans, Barcelona, Spain; Emergency Department, Hospital Universitario 12 de Octubre, Madrid, Spain; Emergency Department, Hospital Universitario 12 de Octubre, Madrid, Spain; Emergency Department, Hospital Universitario de Canarias, Tenerife, Spain; Department of Physical Medicine and Pharmacology, Universidad de La Laguna, Tenerife, Spain; Emergency Department, Hospital Universitario de Torrejón, Madrid, Spain; Faculty of Medicine and Health Sciences, Universidad Nebrija, Madrid, Spain; Toxicology Working Group of the SoCMUE (SoCMUETOx), Universitat Pompeu Fabra, Barcelona, Spain; Vice President of the Spanish Foundation of Clinical Toxicology, Spain; Honorary Professor of Toxicology at the Universitat de Barcelona, Barcelona, Spain; Toxicology Working Group of the SoCMUE (SoCMUETOx), Universitat Pompeu Fabra, Barcelona, Spain; Clinical Toxicology Unit, Emergency Department, Hospital Universitario Son Espases, Palma, Spain; Clinical Toxicology Unit, Emergency Department and Clinical Pharmacology, Hospital Universitario Puerta de Hierro Majadahonda, Madrid, Spain; Faculty of Medicine and Health Sciences, Universidad Nebrija, Madrid, Spain

**Keywords:** cohort studies, emergency service, hospital, lithium poisoning, prognosis, renal dialysis

## Abstract

**Background:**

Lithium poisoning presents as acute, acute-on-chronic, or chronic exposure phenotypes that differ in clinical expression and outcome. The Extracorporeal TReatments In Poisoning Workgroup criteria guide extracorporeal treatment, but whether their prognostic performance varies by poisoning type is uncertain. We assessed these criteria at initial evaluation and whether poisoning type improved risk stratification.

**Methods:**

We conducted a retrospective multicentre cohort study of adult lithium-poisoning episodes between 2015 and 2022 in 26 tertiary hospitals in Spain, Ireland, and Switzerland. Poisoning was defined by compatible exposure plus serum lithium ≥1.5 mEq/l and classified as acute, acute-on-chronic, or chronic. The primary outcome was syndrome of irreversible lithium-effectuated neurotoxicity (SILENT) at 60 days or in-hospital mortality. Sequential logistic-regression models evaluated any criterion, poisoning type, and age; discrimination used the area under the receiver operating characteristic curve (AUC).

**Results:**

Among 644 episodes (136 acute, 196 acute-on-chronic, 312 chronic), the primary outcome occurred in 69 (11%). Any criterion was associated with the outcome after adjustment for poisoning type and age (odds ratio 1.94; 95% confidence interval 1.10–3.44), as were acute-on-chronic poisoning (3.21; 1.27–8.11) and age (per year 1.05; 1.03–1.07). AUC rose from 0.61 to 0.72 with poisoning type and age added. In an exploratory model, the clinical criterion remained associated with the outcome, whereas the analytical criterion did not.

**Conclusions:**

Any Workgroup criterion was associated with adverse outcome. Discrimination improved when poisoning type and age were incorporated, and acute-on-chronic poisoning carried the highest adjusted risk. These findings support interpreting extracorporeal treatment criteria within the clinical context rather than as serum lithium thresholds alone.

KEY LEARNING POINTS **What was known:**

- Extracorporeal TReatments In Poisoning (EXTRIP) criteria guide extracorporeal (dialytic) treatment in lithium poisoning but rest mainly on serum lithium thresholds and renal function.
- Lithium poisoning occurs as acute, acute-on-chronic, or chronic exposure phenotypes with distinct clinical course and presumed prognosis.
- Whether the prognostic performance of these criteria differs by poisoning type was unknown.

**This study adds:**

- In 644 episodes, any EXTRIP criterion at initial assessment was associated with adverse outcome, but discrimination was only moderate.
- Adding poisoning type and age improved prognostic discrimination; acute-on-chronic poisoning carried the highest adjusted risk.
- The clinical, rather than the concentration-based analytical, component carried the prognostic signal.

**Potential impact:**

- EXTRIP criteria may be best interpreted within the clinical context rather than as serum lithium thresholds alone.
- Poisoning type and age could refine early risk stratification and extracorporeal treatment decisions.
- The findings support prospective testing of phenotype- and age-aware dialysis criteria in lithium poisoning.

## INTRODUCTION

Lithium is a cornerstone treatment for bipolar disorder and remains widely used for long-term mood stabilization [1]. However, lithium poisoning continues to be a frequent cause of drug-related toxicity requiring emergency department evaluation [2–4]. The clinical presentation is heterogeneous and depends not only on serum lithium concentration but also on the pattern of exposure and patient-related factors affecting drug distribution and elimination [5–8].

Lithium intoxication may occur as acute, acute-on-chronic, or chronic poisoning, each associated with distinct clinical and prognostic features [9]. Acute intoxication typically follows a large ingestion in patients not previously treated with lithium and is often characterized by early gastrointestinal symptoms with delayed neurologic involvement [10]. In contrast, acute-on-chronic and chronic poisoning occur in patients receiving long-term therapy, in whom neurologic manifestations such as confusion, tremor, and ataxia develop earlier and may be more severe due to prior tissue accumulation of lithium [11, 12]. These differences suggest that the relationship between serum lithium concentrations, clinical manifestations, and outcomes may vary according to poisoning type [12].

The Extracorporeal TReatments In Poisoning (EXTRIP) Workgroup has published consensus recommendations to guide the use of extracorporeal treatment in lithium poisoning [13]. These recommendations rely primarily on serum lithium concentrations, renal function, and clinical severity. However, although the guidelines recognize the existence of different poisoning phenotypes, the extent to which poisoning type influences the prognostic performance of EXTRIP criteria remains uncertain [8, 13, 14].

To address this, we used the SILITOX study, a multicentre cohort of lithium poisoning in emergency departments and clinical toxicology units across three European countries, to evaluate the prognostic value of EXTRIP criteria at initial assessment and whether poisoning type improved risk stratification for adverse outcomes [syndrome of irreversible lithium-effectuated neurotoxicity (SILENT) at 60 days or in-hospital mortality].

## MATERIALS AND METHODS

### Study design and setting

This retrospective, observational, multicentre cohort study collected all episodes of lithium poisoning managed between 2015 and 2022 in the emergency departments and/or clinical toxicology units of 26 tertiary hospitals in Spain, Ireland, and Switzerland. Cases were identified from local records and registries, abstracted from electronic medical records into a pseudonymized database, and reviewed for up to 60 days after the index episode. Data were abstracted locally by one or two site investigators per participating centre, using a common case-report form with prespecified operational definitions for poisoning type, EXTRIP criteria, and outcome ascertainment; abstractors were blind to the specific study hypothesis under evaluation. Data quality was additionally subject to independent external monitoring, performed by a specialist physician member of the coordinating research team.

The study was conducted in accordance with the Declaration of Helsinki and Good Clinical Practice; ethics committees waived informed consent. It was registered in the European Network of Centres for Pharmacoepidemiology and Pharmacovigilance (EUPAS 103542) and follows the STROBE (Strengthening the Reporting of Observational Studies in Epidemiology) statement. It was approved for the Spanish cohort by the Comité de Ética de la Investigación con Medicamentos of Hospital Universitario Puerta de Hierro Majadahonda (EOM 22/23), for the Irish cohort by the SJH/TUH Research Ethics Committee (Project ID 3733), and for the Swiss cohort by the Ethikkommission Nordwest und Zentralschweiz (Project ID 2024-00321).

Race and ethnicity were not systematically recorded in participating centers and were therefore not analysed. The implications of this omission are addressed in the limitations.

### Study population

We included adult patients (aged ≥18 years) evaluated for lithium poisoning, defined by a compatible clinical context (history of lithium exposure or ingestion) together with an initial or peak serum lithium concentration ≥1.5 mEq/l, with or without symptoms. Each patient-episode was treated as an independent unit of analysis. Episodes with insufficient information to characterize the poisoning type or to ascertain primary outcomes were excluded. A small number of episodes assessed during the study period could not be retained in the analytic cohort for this reason, including some lost to clinical follow-up before day 60 without documented recovery or death; exact enumeration by specific exclusion reason was not systematically logged across all 26 participating centres. Ascertainment of the SILENT required clinical follow-up to at least 60 days, documented recovery, or death. Follow-up to day 60 was based on review of the patient’s electronic medical record, and SILENT was adjudicated by the site investigator at each participating centre using the prespecified clinical criteria described in the Outcomes section.

Poisoning episodes were classified as acute, acute-on-chronic, or chronic using definitions consistent with prior literature. Acute poisoning was defined as a single ingestion of lithium in a patient not on chronic lithium therapy. Chronic poisoning was defined as toxicity arising in a patient on established lithium therapy without a discrete additional ingestion, most commonly due to reduced clearance or intercurrent illness. Acute-on-chronic poisoning was defined as an additional lithium ingestion in a patient already on chronic therapy. The selection and classification of episodes are summarized in Fig. S1.

### Data collection and variable definitions

For each episode, we collected demographics (age, sex), comorbidities, characteristics of the index episode (type, presumed cause, co-ingestions, precipitating factors), and clinical manifestations across neurologic, gastrointestinal, and cardiovascular domains. Severity was graded with the Poisoning Severity Score [15]. Laboratory data included initial and peak serum lithium, creatinine, urea, electrolytes, and acid–base variables; initial values were the first at presentation and peak values the maximum during admission. Laboratory data are reported in SI units.

### EXTRIP criteria classification

EXTRIP criteria were applied as defined in the 2015 consensus recommendations for renal replacement therapy in lithium poisoning. We distinguished two criterion types at initial emergency assessment: the analytical criterion (serum lithium ≥ 2.5 mEq/l in chronic or acute-on-chronic poisoning and ≥4.0 mEq/l in acute poisoning) and the clinical criterion (neurologic features meeting the Workgroup threshold, namely, decreased consciousness, seizures, or life-threatening dysrhythmias, regardless of concentration). Each episode was classified at initial assessment into one of four mutually exclusive profiles: no criteria, analytical only, clinical only, or both. The analytical criterion used fixed serum-lithium thresholds and did not incorporate the kidney-function-based modulation of these thresholds described in the EXTRIP consensus recommendations.

### Outcomes

The primary outcome was a composite of SILENT at 60 days or in-hospital mortality. SILENT was defined as persistent neurologic sequelae attributed to lithium toxicity documented at or before 60 days, consistent with published descriptions (cerebellar dysfunction, extrapyramidal syndromes, brainstem dysfunction, or cognitive impairment). Secondary outcomes were SILENT at 60 days and in-hospital mortality, analysed separately.

### Statistical analysis

Continuous variables are summarized as mean (SD) or median (interquartile range) and categorical variables as counts and percentages. Poisoning types were compared with one-way ANOVA or Kruskal–Wallis tests for continuous variables and chi-square or Fisher–Freeman–Halton exact tests for categorical variables.

Agreement between fulfillment of any EXTRIP criterion at initial assessment and actual renal replacement therapy administration was quantified using Cohen’s *κ* statistic with 95% confidence intervals (CIs) (asymptotic method), overall and stratified by poisoning type.

For the primary composite outcome, three sequential multi-variable logistic regression models were fitted: Model 1, any EXTRIP criterion at initial assessment (yes/no); Model 2, plus poisoning type (acute [reference], acute-on-chronic, chronic); Model 3, plus age (per year). Results are odds ratios with 95% CIs. Predictors were specified a priori on clinical grounds rather than by data-driven selection: any EXTRIP criterion as the exposure of interest, poisoning type as a prespecified phenotype hypothesized to modify prognosis, and age as an established prognostic factor reflecting renal reserve, comorbidity, and frailty. This parsimonious set (four degrees of freedom for 69 events) limited overfitting; the mortality model (20 events) was underpowered and is interpreted cautiously. Renal replacement therapy was deliberately not entered as a covariate, as it lies on the causal pathway between criterion fulfilment and outcome and is subject to confounding by indication; the models therefore estimate prognostic associations under prevailing care rather than treatment-independent effects.

The same sequential modelling strategy was applied to the secondary outcomes analysed separately, namely, SILENT at 60 days and in-hospital mortality.

Discrimination was assessed with the area under the receiver operating characteristic curve (95% CI); performance was summarized with the Brier score and calibration with the Hosmer–Lemeshow test, which was uninformative for single-binary-predictor models.

As an exploratory analysis for the primary composite outcome, the overall EXTRIP variable was replaced by its two components, namely, the clinical EXTRIP criterion and the analytical EXTRIP criterion, in an adjusted model including poisoning type and age.

No imputation was performed for missing data, and analyses were conducted using available data for each variable. All tests were two-sided, and *P* < .05 was considered statistically significant. Analyses were performed using IBM SPSS Statistics for Windows, version 26.0 (IBM Corp., Armonk, NY, USA). Outcomes were not formally stratified by sex; given 69 primary events, the study was underpowered to detect sex differences in prognosis.

## RESULTS

### Study population and baseline characteristics

A total of 644 lithium poisoning episodes from 26 centers were included and classified as acute (*n* = 136, 21%), acute-on-chronic (*n* = 196, 30%), and chronic (*n* = 312, 48%) poisoning (Table 1). Baseline characteristics differed across poisoning types, with greater comorbidity burden, lower peak lithium concentrations, and a higher frequency of neurologic manifestations in acute-on-chronic and chronic episodes than in acute poisoning (Table 1).

**Table 1:** Baseline characteristics of lithium poisoning episodes by poisoning type.

Variable	Acute (*n* = 136)	Acute-on-chronic (*n* = 196)	Chronic (*n* = 312)	Total (*n* = 644)
Female sex, *n* (%)	89 (65)	119 (61)	200 (64)	408 (63)
Age, median (IQR), years	54 (39–67)	59 (48–68)	64 (57–72)	61 (51–70)
Age ≥ 65 years, *n* (%)	45 (33)	68 (35)	154 (49)	267 (41)
Hypertension, *n* (%)	37 (27)	82 (42)	132 (42)	251 (39)
Diabetes mellitus, *n* (%)	22 (16)	33 (17)	82 (26)	137 (21)
CKD stage ≥ 2, *n* (%)	7 (5)	9 (5)	37 (12)	53 (8)
Cardiovascular history, *n* (%)	5 (4)	16 (8)	25 (8)	46 (7)
Neurologic history, *n* (%)	15 (11)	29 (15)	47 (15)	91 (14)
Co-ingestion of other toxins, *n* (%)	48 (35)	69 (35)	17 (5)	134 (21)
Initial lithium concentration, mean (SD), mEq/L	2.43 (1.35)	2.36 (1.12)	1.94 (0.59)	2.17 (0.99)
Peak lithium concentration, mean (SD), mEq/L	2.91 (1.42)	2.58 (1.22)	1.97 (0.62)	2.35 (1.10)
Initial creatinine, mean (SD), mg/dL	1.23 (1.04)	1.49 (0.89)	1.46 (0.90)	1.42 (0.93)
Peak creatinine, mean (SD), mg/dL	1.35 (1.13)	1.57 (0.92)	1.52 (0.95)	1.50 (0.98)
Any symptoms, *n* (%)	107 (79)	181 (92)	282 (90)	570 (89)
Digestive symptoms, *n* (%)	51 (38)	96 (49)	81 (26)	228 (35)
Nausea	35 (26)	45 (23)	27 (9)	107 (17)
Vomiting	35 (26)	47 (24)	27 (9)	109 (17)
Diarrhoea	22 (16)	51 (26)	41 (13)	114 (18)
Abdominal pain	5 (4)	19 (10)	21 (7)	45 (7)
Other symptoms	5 (4)	3 (2)	12 (4)	20 (3)
Neurologic symptoms, *n* (%)	89 (65)	158 (81)	270 (87)	517 (80)
Ataxia	17 (13)	25 (13)	78 (25)	120 (19)
Confusion	25 (18)	78 (40)	132 (42)	235 (36)
Decreased level of consciousness	24 (18)	52 (27)	82 (26)	158 (25)
Agitation	14 (10)	29 (15)	36 (12)	79 (12)
Tremor	32 (24)	76 (39)	127 (41)	235 (36)
Myoclonia	2 (2)	22 (11)	23 (7)	47 (7)
Rigidity	2 (2)	25 (13)	23 (7)	50 (8)
Seizures	0 (0)	5 (3)	7 (2)	12 (2)
Cardiovascular symptoms, *n* (%)	14 (10)	30 (15)	30 (10)	74 (11)
Hypotension	10 (7)	23 (12)	21 (7)	54 (8)
Hypertension	4 (3)	6 (3)	7 (2)	17 (3)
QT prolongation	12 (9)	29 (15)	27 (9)	68 (11)
Bradycardia	8 (6)	16 (8)	20 (6)	44 (7)
AV block	1 (0.7)	3 (2)	7 (2)	11 (2)
Poisoning Severity Score ≥2, *n* (%)	49 (36)	110 (56)	134 (43)	293 (46)

Data are presented as *n* (%) unless otherwise specified. Percentages >1% are rounded to whole numbers; percentages <1% are shown to one decimal place. AV, atrioventricular; CKD, chronic kidney disease; IQR, interquartile range; SD, standard deviation.

### Clinical outcomes

The primary composite outcome of SILENT at 60 days or in-hospital mortality occurred in 69 of 644 episodes (11%) and differed by poisoning type, being most frequent in acute-on-chronic poisoning (15%), followed by chronic poisoning (11%) and acute poisoning (4%; *P* = .007) (Table 2).

**Table 2:** Clinical outcomes by poisoning type.

Variable, *n* (%)	Acute (*n* = 136)	Acute-on-chronic (*n* = 196)	Chronic (*n* = 312)	Total (*n* = 644)	*P* value
Composite outcome (SILENT or in-hospital mortality)	6 (4)	30 (15)	33 (11)	69 (11)	.007
SILENT at 60 days	5 (4)	25 (13)	19 (6)	49 (8)	.003
In-hospital mortality	1 (0.7)	5 (3)	14 (5)	20 (3)	.09

*P* values refer to comparisons across acute, acute-on-chronic, and chronic poisoning groups. Percentages >1% are rounded to whole numbers; percentages <1% are shown to one decimal place. SILENT, syndrome of irreversible lithium-effectuated neurotoxicity.

A similar pattern was observed for SILENT at 60 days, whereas in-hospital mortality occurred in 20 of 644 episodes (3%) and did not differ significantly across poisoning types (Table 2).

### Initial assessment according to EXTRIP criteria and subsequent management

At initial assessment, 345 of 644 episodes (54%) met any EXTRIP criterion, predominantly the clinical-only profile. The distribution of EXTRIP profiles differed across poisoning types (Table 3 and Fig. 1).

**Figure 1:**
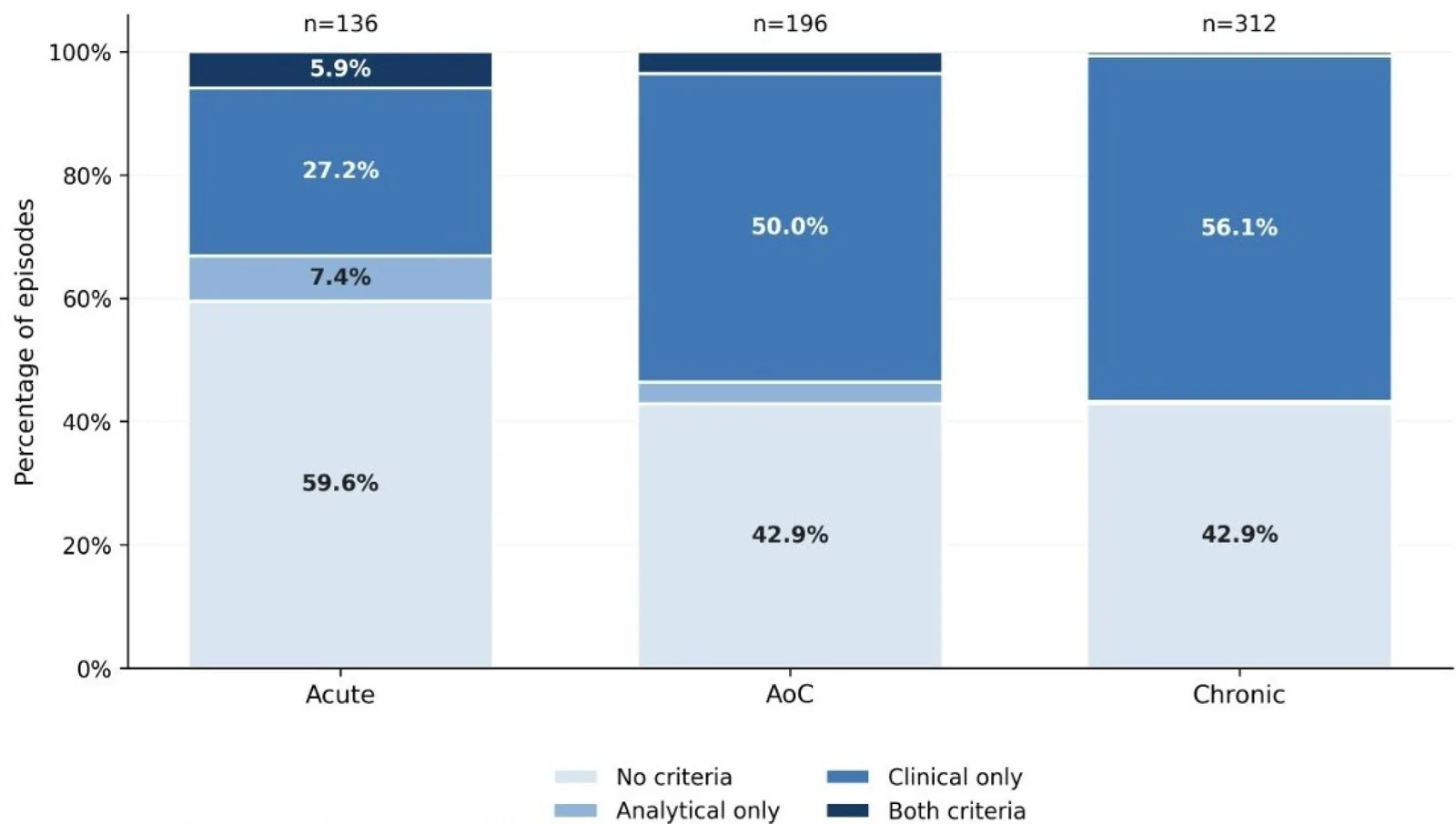
Distribution of EXTRIP profiles by poisoning type. Bars show the proportion of episodes within each poisoning type meeting no EXTRIP criteria, the analytical criterion only, the clinical criterion only, or both criteria at initial emergency assessment. The analytical criterion was a serum lithium concentration ≥2.5 mEq/l in chronic or acute-on-chronic poisoning and ≥4.0 mEq/l in acute poisoning; the clinical criterion was the presence of EXTRIP-defined neurologic features (decreased consciousness, seizures, or life-threatening dysrhythmias) regardless of serum lithium concentration. AoC, acute-on-chronic. EXTRIP, Extracorporeal TReatments In Poisoning Workgroup.

**Table 3:** Initial EXTRIP criteria profile and subsequent renal replacement therapy according to poisoning type.

Variable	Acute (*n* = 136)	Acute-on-chronic (*n* = 196)	Chronic (*n* = 312)	Total (*n* = 644)	*P* value
EXTRIP profile at initial assessment (*P* < .001^a^)					
No EXTRIP criteria, *n* (%)	81 (60)	84 (43)	134 (43)	299 (46)	
Analytical criterion only, *n* (%)	10 (7)	7 (4)	1 (0.3)	18 (3)	
Clinical criterion only, *n* (%)	37 (27)	98 (50)	175 (56)	310 (48)	
Both clinical and analytical criteria, *n* (%)	8 (6)	7 (4)	2 (0.6)	17 (3)	
Any EXTRIP criterion, *n* (%)	55 (40)	112 (57)	178 (57)	345 (54)	
Subsequent management					
RRT performed, *n* (%)	34 (25)	54 (28)	46 (15)	134 (21)	.001
RRT among patients meeting any EXTRIP criterion, *n*/*N* (%)	23/55 (42)	44/112 (39)	37/178 (21)	104/345 (30)	
RRT among patients not meeting EXTRIP criteria, *n*/*N* (%)	11/81 (14)	10/84 (12)	9/134 (7)	30/299 (10)	
Concordance between EXTRIP fulfillment and RRT, %	68	60	52	58	
Cohen’s *κ* (95% CI)	0.30 (0.14–0.46)	0.25 (0.14–0.36)	0.13 (0.06–0.19)	0.19 (0.14–0.25)	

Data are presented as *n* (%) unless otherwise stated. Renal replacement therapy rows are expressed using the relevant subgroup denominator when appropriate. Percentages >1% are rounded to whole numbers; percentages <1% are shown to one decimal place. *P* values refer to comparisons across poisoning types. ^a^Fisher–Freeman–Halton exact test for the distribution of EXTRIP profiles across poisoning types. CI, confidence interval; EXTRIP, Extracorporeal TReatments In Poisoning Workgroup; RRT, renal replacement therapy.

Renal replacement therapy was administered in 134 of 644 episodes (21%) and was more frequent in acute-on-chronic and acute poisoning than in chronic poisoning (28%, 25%, and 15%, respectively; *P* = .001) (Table 3). Among patients meeting any EXTRIP criterion, 104 of 345 (30%) underwent renal replacement therapy, compared with 30 of 299 (10%) among those not meeting EXTRIP criteria. Agreement between EXTRIP fulfillment and renal replacement therapy administration was modest overall (58%; *κ* = 0.19; 95% CI, 0.14–0.25). Agreement was higher in acute poisoning and lower in chronic poisoning (Table 3).

The prognostic implications of EXTRIP profiles also differed across poisoning types. In the overall cohort, no SILENT cases occurred in the analytical-only group, whereas SILENT occurred in 16 of 299 patients (5%) meeting no criteria, 30 of 310 patients (10%) in the clinical-only group, and 3 of 17 patients (18%) in those meeting both criteria. Similarly, the primary composite outcome was most frequent among patients meeting both criteria (4 of 17, 24%) and in the clinical-only group (45 of 310, 15%), while remaining low in the analytical-only group (1 of 18, 6%; *P* = .002) (Table S1).

This pattern was particularly evident among patients who developed SILENT. In acute poisoning, 3 of 5 cases (60%) did not meet the analytical EXTRIP criterion at initial assessment; this occurred in 24 of 25 cases (96%) in acute-on-chronic poisoning and in all chronic cases. Detailed outcome distributions according to EXTRIP profile and poisoning type are shown in Table S1.

### Independent predictors and prognostic performance for the primary composite outcome

Three sequential multi-variable logistic regression models were fitted for the primary composite outcome of SILENT at 60 days or in-hospital mortality (Table 4).

**Table 4:** Multi-variable models and prognostic performance for the primary composite outcome.

Predictor	Model 1—any EXTRIP criterion. OR (95% CI); *P*	Model 2—any EXTRIP criterion + poisoning type. OR (95% CI); *P*	Model 3—Model 2 + age. OR (95% CI); *P*
*N*/events	644/69	644/69	644/69
Any EXTRIP criterion (yes vs no)	2.50 (1.44–4.34); *P* = .001	2.32 (1.33–4.05); *P* = .003	1.94 (1.10–3.44); *P* = .02
Acute-on-chronic vs acute	—	3.47 (1.39–8.65); *P* = .01	3.21 (1.27–8.11); *P* = .01
Chronic vs acute	—	2.26 (0.92–5.56); *P* = .08	1.61 (0.64–4.04); *P* = .31
Age (per year)	—	—	1.05 (1.03–1.07); *P* < .001
Model performance			
AUC (95% CI)	0.61 (0.54–0.67)	0.66 (0.59–0.72)	0.72 (0.67–0.78)
Brier score	0.094	0.093	0.090
Hosmer–Lemeshow *P*	Not applicable	0.69	0.72

Model 1 included any EXTRIP criterion at initial assessment. Model 2 included Model 1 plus poisoning type. Model 3 included Model 2 plus age. Reference categories were acute poisoning and absence of EXTRIP criteria. Area under the receiver operating characteristic curve values are apparent values derived from receiver operating characteristic analysis in the corresponding analytic sample. The Hosmer–Lemeshow test was not applicable for Model 1 because it included a single binary predictor. AUC, area under the receiver operating characteristic curve; CI, confidence interval; EXTRIP, Extracorporeal TReatments In Poisoning Workgroup; OR, odds ratio.

In Model 3, after further adjustment for age, any EXTRIP criterion remained independently associated with the primary outcome (odds ratio, 1.94; 95% CI, 1.10–3.44; *P* = .02). Acute-on-chronic poisoning also remained independently associated with the outcome (odds ratio, 3.21; 95% CI, 1.27–8.11; *P* = .01), whereas chronic poisoning did not (odds ratio, 1.61; 95% CI, 0.64–4.04; *P* = .31). Older age was independently associated with the primary outcome (odds ratio per year, 1.05; 95% CI, 1.03–1.07; *P* < .001).

Model discrimination improved across the sequential models with acceptable calibration in Models 2 and 3 (Table 4). Figure 2 shows the receiver operating characteristic curves for the three sequential models.

**Figure 2:**
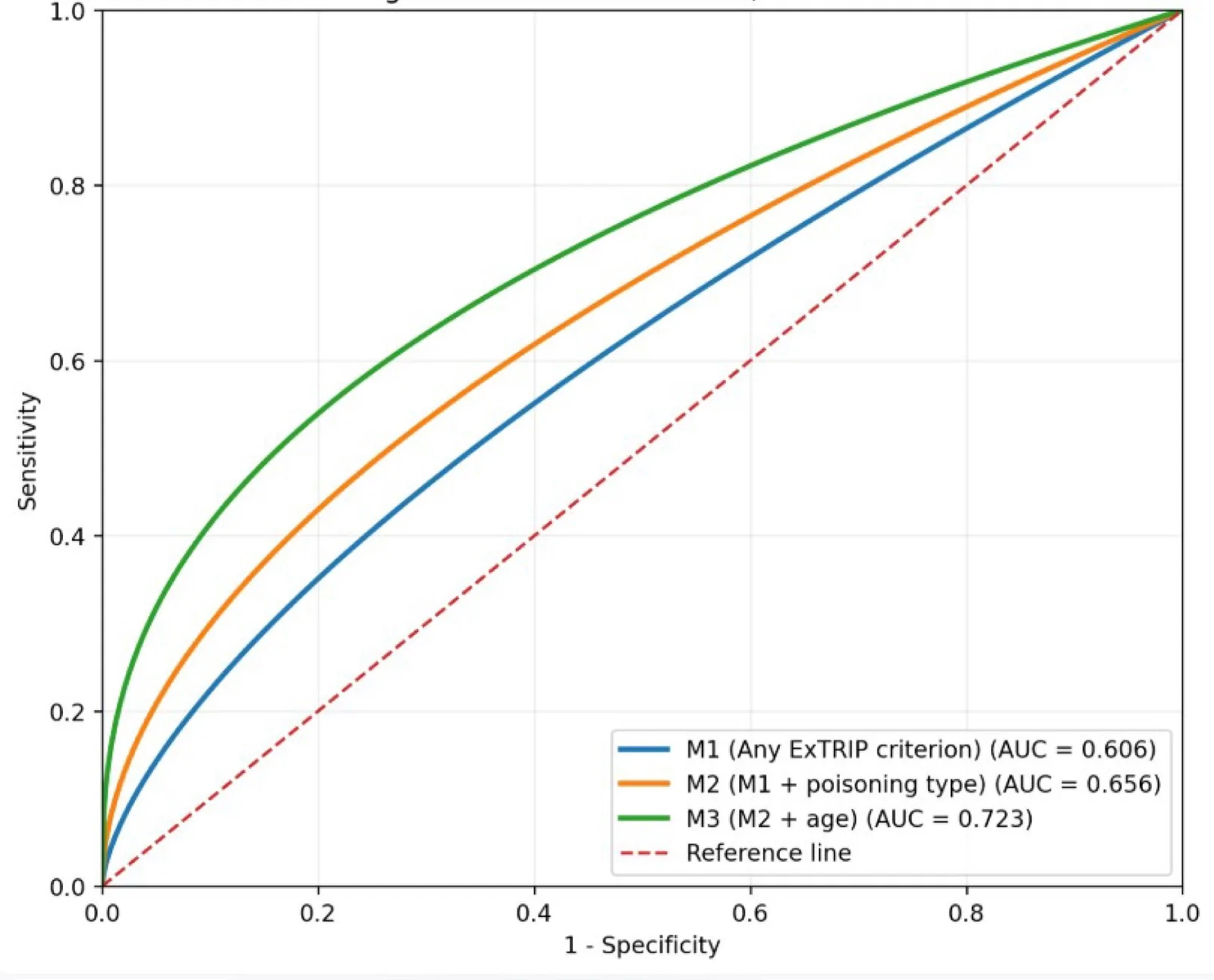
Receiver operating characteristic curves for the sequential prognostic models. Model 1, any EXTRIP criterion; Model 2, Model 1 plus poisoning type; Model 3, Model 2 plus age. Apparent areas under the receiver operating characteristic curve were 0.61 for Model 1, 0.66 for Model 2, and 0.72 for Model 3. The dashed line represents the reference line (AUC = 0.5). AUC, area under the receiver operating characteristic curve; EXTRIP, Extracorporeal TReatments In Poisoning Workgroup.

For the secondary outcomes, the same modelling strategy was applied (Table S2). For SILENT at 60 days, the association with any EXTRIP criterion was attenuated after adjustment and was no longer independently significant in Model 3 (odds ratio, 1.46; 95% CI, 0.77–2.76; *P* = .25), whereas acute-on-chronic poisoning (odds ratio, 3.22; 95% CI, 1.18–8.81; *P* = .02) and older age (odds ratio per year, 1.05; 95% CI, 1.03–1.08; *P* < .001) remained independently associated. For in-hospital mortality, any EXTRIP criterion remained independently associated across all three models, including Model 3 (odds ratio, 4.01; 95% CI, 1.15–14.03; *P* = .03), whereas poisoning type and age were not independently associated.

In an exploratory adjusted model replacing the overall EXTRIP variable with its clinical and analytical components, the clinical EXTRIP criterion remained independently associated with the primary composite outcome (odds ratio, 2.03; 95% CI, 1.15–3.57; *P* = .02), whereas the analytical criterion did not retain an independent association (odds ratio, 2.21; 95% CI, 0.75–6.52; *P* = .15).

## DISCUSSION

In this multicentre cohort of lithium poisoning presenting to emergency departments, any EXTRIP criterion at initial assessment was independently associated with the primary composite outcome, and discrimination improved when poisoning type and age were added. Acute-on-chronic poisoning consistently showed the highest risk, whereas chronic poisoning lost independent significance after adjustment. The prognostic signal related more to the clinical than to the analytical component, indicating that EXTRIP criteria should be interpreted within the context of poisoning phenotype and age rather than as serum lithium thresholds alone.

Fulfilment of any EXTRIP criterion at initial assessment carried prognostic information, supporting its use as an early risk marker. However, the moderate discrimination of the EXTRIP-only model indicates that the criteria do not fully capture prognostic heterogeneity. Discrimination improved once poisoning type and then age were added, suggesting that the signal becomes more meaningful when interpreted within the context of exposure pattern and patient vulnerability.

Among phenotypes, acute-on-chronic poisoning showed the most consistently unfavourable prognosis, robustly across the sequential models for the composite outcome and for SILENT. This is clinically plausible, as acute-on-chronic poisoning may combine the toxic burden of recent ingestion with preexisting tissue lithium accumulation and reduced physiological reserve. In contrast, chronic poisoning lost independent significance after adjustment, suggesting that its poorer crude outcomes are explained partly by age and clinical severity rather than chronicity alone.

These differences mirror the distinct pharmacokinetics of lithium toxicity. In acute poisoning, lithium is initially confined to the extracellular compartment, so neurotoxicity may be delayed until redistribution into the central nervous system [10, 16, 17]. In chronic therapy, tissue compartments including the brain are already saturated, so even modest rises in serum concentration may cause significant neurotoxicity [12, 18]. This helps explain why acute-on-chronic poisoning carried the highest risk and why many patients who developed SILENT did not meet analytical thresholds at initial assessment, supporting serum concentration as an imperfect marker of neurotoxic risk in which clinical context is central [14].

Older age was also a strong, independent predictor of the composite outcome and of SILENT. This is coherent, as older patients may have lower kidney reserve, greater frailty and comorbidity, and less tolerance of severe neurotoxicity. Because age improved discrimination, it should inform early interpretation of poisoning severity even when EXTRIP criteria are considered.

A particularly relevant aspect of our findings is the apparent difference between the clinical and analytical components of EXTRIP criteria. In the exploratory adjusted model, the clinical EXTRIP criterion remained independently associated with the primary composite outcome, whereas the analytical criterion did not. This does not mean that serum lithium concentration is unimportant; rather, it suggests that lithium thresholds alone may incompletely reflect the true biological and neurologic impact of poisoning. Serum lithium concentration is only one dimension of toxicity and may be influenced by timing of measurement, redistribution, chronic exposure, and the mismatch between serum and tissue burden. In contrast, clinical manifestations likely reflect the integrated expression of toxicity at the organ level and may therefore provide a more direct signal of adverse outcome. This interpretation is consistent with previous observational studies suggesting that the clinical component of EXTRIP criteria may be more informative than concentration-based thresholds alone [7, 14].

This is reinforced by the secondary outcomes. For SILENT at 60 days, the association with any EXTRIP criterion attenuated and lost significance after full adjustment, whereas acute-on-chronic poisoning and age persisted, indicating that longer-term sequelae are shaped by host vulnerability and phenotype as much as by initial fulfilment. For in-hospital mortality, any EXTRIP criterion remained associated across all models, indicating a closer link to short-term severe outcomes than to delayed sequelae.

The modest agreement between any EXTRIP criterion and actual renal replacement therapy likely reflects the complexity of real-world decisions: clinicians weigh trajectory, response to supportive care, comorbidity, haemodynamic stability, and perceived reversibility, and some patients meeting criteria improve before dialysis or are judged manageable without it [19]. Because renal replacement therapy was not modelled (see Materials and methods section), this discordance also has an analytic implication: criterion fulfilment and dialysis are entangled in routine care, so the estimates reflect outcomes under heterogeneous treatment rather than untreated natural history, and residual confounding by indication cannot be excluded. EXTRIP criteria therefore function better as a structured risk framework than as a proxy for real-world dialysis use.

These findings have practical implications for emergency physicians, nephrologists, and toxicologists: any EXTRIP criterion should flag increased risk, but that risk should be refined by poisoning type and age, with particular caution in acute-on-chronic poisoning, and read through the clinical expression of toxicity rather than lithium thresholds alone.

This study has the following strengths: to our knowledge it is the largest multicentre European cohorts evaluating EXTRIP criteria in lithium poisoning across acute, acute-on-chronic, and chronic presentations, applied at initial emergency evaluation, with clinically meaningful outcomes including SILENT at 60 days. Companion analyses of this cohort separately derived a pragmatic model to predict SILENT [20] and examined renal replacement therapy relative to EXTRIP criteria [21], which the present prognostic analysis complements.

Several limitations should be acknowledged. The retrospective design may have produced incomplete or heterogeneous recording across centres, and phenotype classification, though clinically meaningful, may be misclassified in borderline cases. Some outcomes, especially in-hospital mortality (20 events), were infrequent, limiting precision. Because management was not protocolized, the relationship between EXTRIP fulfilment and renal replacement therapy reflects real-world practice and may be influenced by centre-level variation, clinician judgement, and confounding by indication. The cohort was underpowered for sex differences and was not sex-stratified, missing data were not imputed, and the comparison of clinical versus analytical components was exploratory and hypothesis-generating. Data abstraction relied on one or two investigators per centre without a formal double-abstraction design or a quantitative inter-rater reliability estimate for poisoning-type classification or SILENT ascertainment; abstractors were, however, blind to the specific study hypothesis, and data quality was subject to independent external monitoring, so residual misclassification cannot be fully excluded. We also did not incorporate the kidney-function-graded modulation of the EXTRIP analytical threshold, which lowers the recommended concentration cut-off when kidney function is significantly impaired; because age, already incorporated and independently prognostic, captures much of the variance in baseline kidney reserve, we judged that a separate, retrospectively-graded renal-function criterion could not be reliably operationalized in this cohort. In addition, exact enumeration of episodes excluded because the primary outcome could not be ascertained, including the number lost to clinical follow-up before day 60, was not systematically logged across all 26 participating centres and could not be retrospectively reconstructed; Fig. S1 therefore summarizes only the composition of the final analytic cohort rather than the pre-inclusion screening and exclusion process.

In conclusion, in this large multicentre cohort, any EXTRIP criterion at initial assessment was associated with adverse outcome, but discrimination improved when poisoning type and age were incorporated. Acute-on-chronic poisoning showed the highest risk, and the prognostic signal related more to clinical manifestations than to serum lithium thresholds alone, supporting integration of phenotype and age into the early interpretation of EXTRIP criteria.

## Supplementary Material

sfag328_Supplemental_File

## Data Availability

The datasets generated and analysed during the current study are available from the corresponding author upon reasonable request and subject to applicable ethics and data protection requirements.
