# Supplementary material for: Clinical context enhances interpretation of extracorporeal treatment criteria in lithium poisoning
Source: Clin Kidney J. 2026 Sep 24;19(10):sfag328. doi: 10.1093/ckj/sfag328 (PMC13659238; doi:10.1093/ckj/sfag328)
Supplement: Supplementary file 1 [file sfag328_supplemental_file.pdf]

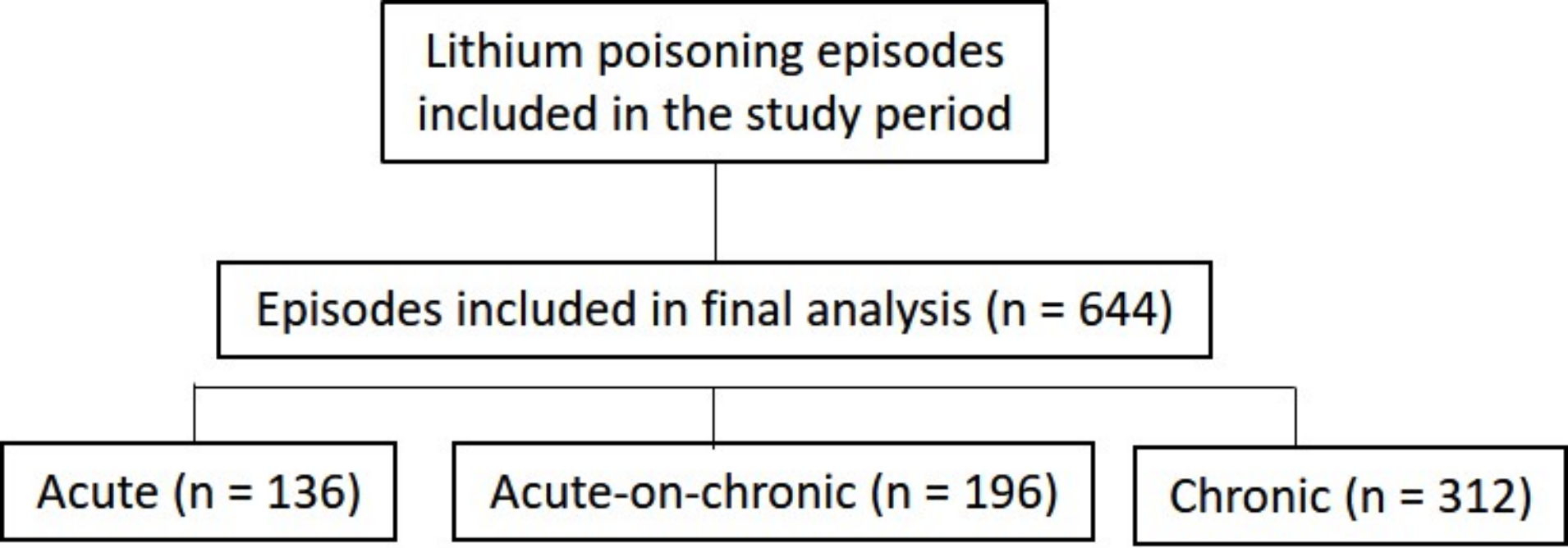
Lithium poisoning episodes included in the study period
Episodes included in final analysis (n = 644)
Acute (n = 136)
Acute-on-chronic (n = 196)
Chronic (n = 312)
